# Temporomandibular pain and quality of life assessment in adolescents in a Norwegian cohort

**DOI:** 10.1002/cre2.733

**Published:** 2023-05-26

**Authors:** Anne F. Dahl, Anne K. Bergem, Tore Bjørnland, Heming Olsen‐Bergem

**Affiliations:** ^1^ Sykehuspartner HF Oslo Norway; ^2^ Psychiatric Division The Norwegian Medical Association Oslo Norway; ^3^ Department of Oral Surgery and Oral Medicine, Faculty of Dentistry, Institute of Clinical Dentistry University of Oslo Oslo Norway

**Keywords:** adolescents, pain, quality of life, temporomandibular joint

## Abstract

**Objective:**

The objective was to examine the prevalence of pain from the face and temporomandibular joint (TMJ) and oral function in adolescents and contribute to more focus on this patient group.

**Methods:**

A total of 957 adolescents were included in this study, in age cohorts 18, 16, and 14, scheduled for a dental recall examination. Clinical data were collected as a part of the routine clinical examination. All participants also answered a survey.

**Results:**

Almost half of the participants had experienced facial pain in the last 3 months, headache being the most prevalent site reported. A significantly higher prevalence was found for females for all pain sites, and facial pain was significantly higher among the oldest. A reduced maximal incisal opening was significantly associated with higher reported facial/jaw pain, with increased mouth opening pain and chewing pain. Fifty‐seven percent of the participants reported the use of nonprescription painkillers, highest among females, and in the oldest age cohort, mainly caused by nonfeverish headaches. General health was found to be negatively correlated to facial pain, headache, pain intensity, and duration, pain upon oral function, and oral movement, as well as the use of nonprescriptive drugs. Females in the older age group, experience less quality of life in general, as they felt more worried, anxious, lonely, and sad, compared to males.

**Conclusion:**

Facial‐ and TMJ pain was higher in females, and higher with increasing age. Almost half of the participants had experienced facial pain in the last 3 months, headache being the most prevalent site reported. General health was found to be negatively correlated to facial pain.

## 1 INTRODUCTION

Temporomandibular disorder (TMD) is a common term used to describe dysfunction and pain affecting masticatory muscles and/or the temporomandibular joint (TMJ) with its surrounding structures. TMD can be divided into extra‐ and intra‐articular conditions. Myofascial pain belongs to the extra‐articular conditions, while disk displacement, arthrosis, and arthritis belong to the intra‐articular conditions (Bjørnland & Møystad, 2010; Gauer & Semidey, 2015). The three major signs and symptoms of TMD, are pain, limited range of motion, and TMJ sounds (List & Jensen, 2017; Scrivani et al., 2008). In cases with TMD pain, most adults and adolescents also report headaches (Ciancaglini & Radaelli, 2001; List & Jensen, 2017). Patients with myalgia are more likely to have bilateral symptoms compared with patients with arthrosis and disc displacement (Bjørnland & Møystad, 2010).

Müller et al. (2013) did a large study on 20,719 children and adolescents (age range 4–17 years) that showed the mean maximal mouth opening capacity (MOC) was 45 mm.

Landtwing (1978) recorded MOC in children and adolescents and observed a slight increase in the median value from 43 to 55 mm for 5‐ and 18‐year‐olds, respectively.

A study by Köhler et al. (2009) showed that up to 50% of children and adolescents had at least one sign of TMD, but only 1%–2% needed treatment. Moyaho‐Bernal et al. (2010) found that the prevalence of TMD is particularly high in children with mixed dentition and that muscular symptoms dominate. List et al. (1999) found a 7% prevalence of TMD pain in children and adolescents and reported the prevalence of TMD‐related pain was significantly higher in females than males.

Recently two studies of TMD in children and adolescents have been implemented in Norway. Graue et al. (2016) found a prevalence of 7.2% among adolescents in Bergen County based on two self‐reported screening questions for TMD‐related pain. The following clinical examination based on the diagnostic criteria (DC)/TMD criteria for TMD, suggested a higher prevalence of 11.9%, with a peak at 16 years. The two screening questions Graue et al. (2016) used, confirmed very good reliability and high validity by Nilsson et al. (2005, 2006) Another Norwegian study (Østensjø et al., 2017) found a prevalence of 7% among adolescents in Rogaland County based on the same two self‐reported questions pertaining to painful TMD‐P (Nilsson et al., 2005, 2006) and clinical examination to support the findings.

Several studies of the adult population show that TMD can be associated with reduced general health, depression, and other psychological disabilities, and may affect the quality of life for the patient (Bjørnland & Møystad, 2010; Gauer & Semidey, 2015; List & Jensen, 2017; Scrivani et al., 2008). Wahlund (2003) found that adolescents with TMD‐related pain reported higher absences from school and higher consumption of analgesics compared to the control group. In addition, TMD seems to have a strong association with stress, somatic complaints, and emotional problems in adolescents (Al‐Khotani et al., 2016; Wahlund, 2003). Persistent pain in children and adolescents has been associated with reduced quality of life (Haraldstad et al., 2017).

The aims of this study were to examine the prevalence of pain from the face and TMJ and oral function in adolescents and contribute to more focus on the patient group with this type of pain for faster intervention.

## 2 MATERIALS AND METHODS

### 2.1 Design and setting

**Figure 1:**
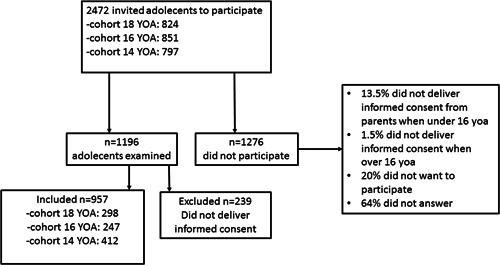
Flowchart for the study population. YOA, years of age.

This clinical cross‐sectional cohort study was conducted from September 2019 to March 2020 in Vestfold County, Norway. All adolescents in age cohorts 18, 16, and 14 years of age (YOA) (*n* = 2472), scheduled for a dental recall examination, were invited to participate in the study. Of these, 957 adolescents (485 females, 472 males, 412 YOA 18, 247 YOA 16, 298 YOA 14) were included in the study (Figure 1).

All public dental clinics in Vestfold County, counting 15, participated in the study. Ten of the dental clinics are in bigger municipalities with more than 25,000 residents. This applies to Tønsberg, Sandefjord, Larvik, Horten, and Færder. Five dental clinics are in a smaller municipality with less than 15,000 residents. This applies to Sande, Svelvik, Holmestrand, and Re. Some of the municipalities have up to three dental clinics. The population figures are taken from Statistics Norway (2020) from the end of 2019.

In 2019, there were around 210,000 residents in Vestfold, of which 58% were employed and 1% were registered as unemployed. The most common reason for not being in work or looking for work was age, 19.6% of the population in Vestfold over the age of 15 were old‐age pensioners or had retired with a contractual pension. Compared to other counties, Vestfold had a high proportion of people not working due to health‐related reasons, 8.4% of the population over the age of 15 received unemployment benefits or disability benefits (Statistics Norway, 2023a). From 2018 to 2020, 14% of children and adolescents in Vestfold, at the age 0–17 years old, lived in households with a permanent income below 60% of the country's/county's/municipality's median income, calculated according to the EU scale (Kommunehelsa statistikkbank, 2023).

In 2019, 13% of the population in Vestfold were immigrants, Sandefjord with the highest proportion of immigrants of the municipalities in Vestfold. In addition, 3% of the population were Norwegian‐born with immigrant parents (Statistics Norway, 2023b).

All participants were examined at their own clinic, where the patients were familiar with the staff. Fifteen clinics conducted the examinations. All examiners were calibrated by the two project leaders, ensuring the same clinical examination, equal measures of function and pain, and a calibrated palpation pressure of 1 km. All the dental clinics were brought together for a review of how the patients were to be examined for this project, both with regard to palpation of muscles and measurement of jaw movements. The examiners received a detailed written description explaining pictures of where and how the various muscle groups should be palpated. In addition, there was made two video presentations, one where the palpation of muscles was shown and one where the measurement of jaw movements was shown. To ensure equal palpation force, the examiners also were told to practice reproducing 1 kg pressure on weight, for the most correct palpation possible. All participants answered a survey on a digital pad, whereas clinical data were recorded both in Questback (2020) and in the standard journal system.

Inclusion criteria:

– Subjects belonging to public dental supervision from Dental Service in Vestfold County.
– Their normal routine check at their local dental clinic was in the period September 1, 2019 to February 28, 2020.
– Males and females represented cohorts of 2002 (18 YOA), 2004 (16 YOA), and 2006 (14 YOA).
– Declaration of consent from parents when the age of subjects were below 16 years old at the examination date.

Exclusion criteria:

– Subjects belonging to private dental supervision.
– Subjects not meeting routine examinations in the specific period.
– Subjects with caries to a pulp last 3 months.
– Subjects exposed to head and neck trauma last 3 months.
– Subjects being ill with a fever last 3 months.

There was no additional control group included. If there was any need for treatment, this was performed at regular appointments at the dental clinic. Of the whole group of adolescents examined, 957 (80%) were included in the study (Figure 1). Drop‐out statistics were performed, and data from the 20% of adolescents who were examined, but not included, showed no differences. Late drop‐out was mainly because the alleged participants had moved, was unable to answer due to disease, disability, or language problems, or lacked written consent.

### 2.2 Data collection

Both the clinical examination and the questions in the survey were based on the questionnaire and the clinical examination of the validated diagnostic tool DC/TMD (Schiffman et al., 2014). The questions related to face and jaw pain were based on DC/TMD, Graded Chronic Pain Scale (chronic pain intensity subscale, version 2), and Patient Health Questionnaire‐4 (emotional functioning) (Schiffman et al., 2014) The questions regarding the quality of life were taken from the measurement system from Directorate of Health/National Institute of public health on life quality, all questions on the minimum list of subjective life quality were included in the survey, measuring subjects such as life satisfaction, affects and eudaimonic quality of life (i.e., meaning and purpose) (Bang Nes et al., 2018).

### 2.3 Clinical examination

Clinical data were collected as a part of the routine clinical examination, which contained a calibrated palpation of face‐ and jaw muscles with the pressure of 1 km and jaw movements such as maximal incisal opening (MIO) and laterotrusion of the mandible to both sides. MIO and laterotrusion movement were measured with a ruler during the clinical examination and given in millimeters. MIO is the greatest distance between the incisal edge of the maxillary central incisor to the incisal edge of the mandibular central incisor when the mouth is opened as wide as possible. Maximal laterotrusion was measured the same way, only with the mandible maximally shifted to each side.

Pain symptoms were evaluated at baseline with a visual analog scale (VAS) from 0 to 10, where 0 was no pain and 10 was the worst imaginable pain. The specific muscle/joint‐related clinical examination was based on the validated tool DC/TMD, axis I. The DC/TMD decision tree was not used within this study, and the exclusion of other reasons for joint pain, headache, and myalgia was not performed within the scope of this study, which was performed as a single examination only. Hence, the patients were not fully diagnosed according to DC/TMD, and no certain diagnosis of arthritis, TMD headache, or myalgia was obtained. In accordance with (Nilsson et al., 2006), a TMD diagnosis requires one or two positive answers to questions about at least weekly pain in the face, the temporal area, the TMJs, and/or upon mouth opening or chewing.

### 2.4 Data analysis

Quantitative data were transferred from Questback (2020) to Excel and SPSS (IBM SPSS Statistics version 27.0.0.0/www.ibm.com). Descriptive analysis and correlation statistics were performed (Spearman and Pearson). For between‐group comparisons, nonparametric analysis (Mann–Whitney and Kruskal–Wallis) was performed, as well as one‐way analysis of variance. All the tests were carried out at a confidence level of 95%, and probabilities of 0.05 or less were accepted as significant.

### 2.5 Ethical considerations

This study was approved by the Norwegian Regional Committees for Medical and Health Research Ethics and Norwegian Centre for Research Data, reference number 2018/1615.

## 3 RESULTS

### 3.1 Oral function

The mean MIO was 48.8 mm, with a small gender difference in males (mean 49.9 mm) and females (mean 47.5 mm). A reduced MIO was significantly associated with higher reported facial/jaw pain last 3 months (*p* = .001), with increased mouth opening pain and chewing pain (*p* = .04), with reported VAS pain intensity (*p* = .05), and with palpation pain of masseter muscle (*p* = .02). MIO was also significantly decreased in adolescents with high VAS self‐assessed masseter muscle palpation pain (*p* = .01), but not with other chewing muscles, and not with masseter muscle pain during the clinical examination. It was appreciated a positive significant correlation between MIO and gender (males), and MIO and lateral movement, whereas a negative significant correlation was observed between MIO and facial/jaw pain last 3 months, MIO and pain intensity, MIO and the number of days with pain, MIO and pain upon opening, and MIO and chewing pain. Further 33 participants (3%) had MIO less than <37 mm with 14 mm as the lowest number and 521 participants (54%) had MIO in a range of 40–50 mm. Of the participants, 343 (36%) had MIO above 50 mm, and of those 73 participants (8%) had a range of 60–65 mm. Lateral movement was more clustered around the mean, with a mean of 10.5 mm, and few in the low‐/high‐end range. One participant was unable to understand how to move the jaw sideways, whereas 10 had lateral movement below 7 mm. Thirty‐nine participants had a lateral movement range of 15–20 mm. There was no difference in gender or age cohorts regarding lateral movement (Tables 1a and 1b). Pain upon palpation of masseter muscle was significantly associated with limitation of lateral jaw movement to the same side (*p* = .001), as well as to the contralateral side as palpated (*p* = .04).

**Table 1a:** Mouth opening and lateral movement.

	Mean (*n* = 957), mm	Minimum, mm	Maximum, mm	Mean F (*n* = 485)	Mean M (*n* = 472)
MIO	48.8	14	65	47.5	49.9
Lat L	10.5	0	20	10.4	10.5
Lat R	10.4	0	20	10.3	10.4

Abbreviations: F, female; L, left; Lat, lateral movement; M, male; MIO, maximum incisal opening; R, right.

**Table 1b:** Mouth opening and lateral movement in age cohorts.

	Mean, mm	Minimum, mm	Maximum, mm
18 YOA *n* = 412			
MIO	49.3	15	65
Lat L	10.7	0	20
Lat R	10.3	0	20
16 YOA *n* = 247			
MIO	49.4	31	65
Lat L	10.7	1	20
Lat R	10.7	1	20
14 YOA *n* = 298			
MIO	47.5	14	65
Lat L	10.7	0	20
Lat R	10.3	0	20

Abbreviations: L, left; Lat, lateral movement; MIO, maximum incisal opening; R, right; YOA, year of age.

Of the participants, 18.7% (*n* = 179) reported restricted MOC during the last month. Of these, 2.5% (*n* = 24) experienced this quite often or often. This problem was more frequent in females (4.1%) than males (0.8%), and in 18‐year‐olds (3.4%) versus the 14 YOA (1%). Clicking from the TMJ was reported in 47.1% (*n* = 451), reported as quite often in 6.2% (*n* = 59), and often in 4.8% (*n* = 46), with a significant difference between females (8.2% quite often, 6.2% often) and males (4% quite often, 3.4% often). The age difference was also reported, with significantly higher frequencies in 18 YOA versus 14 YOA (Table 2).

**Table 2:** Self‐reported health, oral health, pain, and general mental health status.

		0—very bad (in %)	1—bad (in %)	2—neither good nor bad (in %)	3—very good (in %)	Did not answer
How is your health?	A	0.5/0.6/0.4	2.7/4.1/1.3	35.9/37.9/33.8	56.9/52.9/60.9	3.9/4.5/3.6
	B	1/0/0.3	4.9/1.6/0.7	39.1/36.8/30.9	51.2/57.1/64.8	3.9/4.5/3.4

		**0**—**not at all (in %)**	**1**—**some days (in %)**	**2**—**most days (in %)**	**3**—**nearly every day (in %)**	**Did not answer**
Decreased interest in or the joy of doing things?	A	32.8/27.2/38.5	48.8/51.2/46.1	7.9/9.7/6.1	6.6/7.6/5.5	3.9/4.3/3.8
	B	29.9/31.2/38.3	50.7/49.8/45.3	10.2/7.7/5	5.1/6.9/8.4	4.1/4.5/3

		**0**—**not at all (in %)**	**1**—**some days (in %)**	**2**—**most days (in %)**	**3**—**nearly every day (in %)**	**Did not answer**
Felt down, depressed or filled with hopelessness	A	50.7/37.7/63.8	38.1/46.9/29	5.1/7.6/2.5	2.2/3.3/1.1	3.9/4.5/3.6
	B	43.4/48.2/62.8	42.5/39.7/30.9	6.1/6.5/2.7	4.1/0.8/0.7	3.9/4.9/3

		**0**—**never (in %)**	**1**—**rarely (in %)**	**2**—**sometimes (in %)**	**3**—**quite often (in %)**	**4**—**often (in %)**	**Did not answer**
Experienced facial pain last 3 months?	A	52.9/47.3/58.4	32.5/32.3/32.6	10.9/14.6/7	2.6/3.7/1.5	0.9/1.6/0.2	0.2/0.4/0.4
	B	49.8/50.2/59.4	33.7/36.4/27.5	10.9/10.5/11.1	3.4/2.4/1.7	2.2/0/0	0/0.5/0.3
Experienced a headache last month?	A	25.3/18.9/31.7	31.6/27.4/35.7	27.3/31.5/22.8	8.9/13.8/3.8	3.9/5.8/1.9	3.1/2.7/4
	B	20.9/24.3/32.2	28.6/31.6/35.6	32.3/27.9/19.8	10.9/8.9/6	4.4/4.9/2.3	2.9/2.4/4
Pain upon mouth opening last month?	A	75.9/73.5/78	15.5/14.6/16.3	5.6/7.2/4	2/2.9/1.1	0.7/1.4/0	0.3/0.4/0.6
	B	72.1/77.3/79.9	17/13.8/14.8	6.8/6.1/3.7	2.7/2/1	1.5/0/0.3	0/0.8/0.3
Pain upon chewing hard food last month? (carrot/apple, etc.)	A	62.2/58.4/65.8	24.2/25.7/22.6	10.4/11.3/9.5	2.2/3.1/1.3	0.6/1/0.2	0.3/0.4/0.6
	B	61.4/65.2/60.7	24.5/22.7/25.2	10.4/9.7/11.1	2.4/1.6/2.3	1.2/0.4/0	0/0.4/0.7
Problems with full mouth opening capability last month?	A	81.3/78.8/83.5	10.6/10.1/11	5.5/6.8/4.2	1.4/2.5/0.2	1.1/1.6/0.6	0.1/0.2/0.4
	B	78.4/83.4/83.6	10.9/9.7/10.7	7.3/3.6/4.7	1.5/2/0.7	1.9/0.8/0.3	0/0.4/0
Clicking from TMJ during last year?	A	52.9/48.4/57.3	21.2/19.5/22.8	14.6/17.3/11.8	6.2/8.2/4	4.8/6.2/3.4	0.3/0.4/0.6
	B	46.4/54.3/60.7	20.6/20.6/22.5	16.3/14.2/12.8	9.2/5.3/2.7	7.3/4.9/1.3	0/0.8/0
Used nonprescriptive painkillers last 3 months (not menstruation pain)?	A	42.9/36.8/49	32.6/36/29	14.4/14.6/14.2	4.3/6.4/2.1	5.3/6/4.7	0.4/0.2/1.1
	B	34/41.3/56.7	32.8/35.2/30.2	18.9/13.8/8.7	6.1/4/2	8.3/4.9/1.7	0/0.8/0.7

*Note*: Total number of participants: 957; 485 females and 472 males. Cohorts: 18 YOA = 412, 16 YOA = 247, and 14 YOA = 298. The results are presented in line A; the total number of participants/females/males, line B; 18 YOA/16 YOA/14 YOA, with graded responses from 0 to 3 or 4. All numbers are presented as % of the cohort.

Abbreviations: TMJ, temporomandibular joint; YOA, year of age.

### 3.2 Pain

Among the participants, 47% (*n* = 450) did experience facial pain in the last 3 months. Of these, 3.5% experienced facial pain quite often or often. Considering gender, facial pain was significantly more frequent in females (5.3%) as compared to males (1.7%). Facial pain also increased significantly with age, 5.6% in the oldest cohort, versus 1.7% in the youngest age group.

*Mouth opening pain* was present last month in 24% (*n* = 230), with 2.7% reporting it to be quite often/often. Again, pain is more frequent upon mouth opening in females (4.3%), and in 18‐year‐old (4.2%).

*Chewing pain* last month was reported in 37.8% (*n* = 362), but most rarely or sometimes, quite often/often was reported in 2.8% (4.1% in females, 3.6% in 18 YOA). There was no correlation between pain upon chewing and current orthodontic treatment.

*Headache* is the most frequent pain experience last month (74.7%, *n* = 715), and 12.7% (*n* = 122) experienced headaches quite often/often. Headache was significantly higher in females (19.6%) versus males (5.7%) and was significantly lower in the youngest age group (8.3% in 14 YOA, 13.8% in 16 YOA, 15.3% in 18 YOA).

There was no correlation between headache and mouth function (MIO and lateral movement). More closely, 57% (*n* = 546) of the participants used nonprescription painkillers (paracetamol/acetaminophen 500 mg, Ibuprofen 200 mg, and 400 mg) the last 3 months, medication to prevent menstrual pain/cramps were excluded, and 4.3% used painkillers often, mostly females, and in the oldest age cohort (Table 2). Reported use for these drugs was due to injury and fever, but the main reason was due to nonfeverish headache (*n* = 309).

Among the approximately 2% of adolescents reporting often (every day or every other day) or quite often (several times a week) pain upon mouth opening, almost all participants reported pain upon chewing, and there was also a strong correlation with a headache. In this group, there was a high frequency of TMJ clicking, although no closed lock was found. However, there is neither any reduced movement of the jaw compared to the rest of the participants, nor any differences when comparing the use of nonprescription painkillers. Life quality was not correlated with any of these observations. Adolescents reported good general health, good life quality, and meaningful life.

No participants reported pain specifically from the TMJ, and no pain upon palpation of the TMJ was recorded. Table 3 describes the degree of pain upon palpation of muscles in females and males. Females expressed somewhat higher palpation pain (21.3%), compared to males (15.5%). In females, palpation pain of the temporal muscle was most painful, whereas the masseter muscle (at jaw angle) was most painful in males. Both genders expressed relatively high pain levels upon palpation of the sternocleidomastoid muscle. In accordance with the criteria from Nilsson et al. (2006) in this study, 11 patients had TMD.

**Table 3:** Palpation of muscles in 957 participants with 472 males and 485 females in the ages groups 14–18 years of age.

	Males (0/1/2/3)	Females (0/1/2/3)
m.masseter jaw angle	80.7/15.2/4/0.1	77.6/16.8/4.8/0.8
m.masseter upper part	83.2/13.3/3.2/0.3	78.5/16/4.7/0.8
m.temporalis	82.9/13.7/3.1/0.3	75.9/16.8/6.3/1
m.sternocleidomastoid posterior ear region	84.4/11.3/3.9/0.4	78.8/15.2/4.9/1.1
m.sternocleidomastoid chest region	87.8/9.3/2.7/0.8	81.7/14/3.7/0.6
neckmuscles	87.8/8.4/3.7/0.1	80/15.9/3.6/0.5

*Note*: The results are presented with gradings; 0 = no pain upon palpation/1 = express pain upon palpation/2 = express pain and involuntary eyebrow movement/3 = express pain and movement away from the palpation point. All numbers in % of the total number of participants and as a mean of palpation of the left and right side.

### 3.3 General health

Very bad or bad health was reported by 31 adolescents (3.2%), but 56.9% (*n* = 545) reckoned their health to be very good, especially among males.

Regarding general interest in or the joy of doing things, 14.5% (*n* = 139) felt a decreased interest, somewhat higher in females (17.3%) versus males (11.6%).

Of the adolescents, 69.9% (*n* = 669) were happy with their life at present, and 66.2% (*n* = 634) felt mainly happy last week, whereas 59.1% (*n* = 566) experienced life as very meaningful. However, there were large discrepancies between genders and in different age groups, as males and 14‐year‐olds scored higher on both life happiness and meaningfulness. This is a general reflection in this population, as females and the oldest age group express a significantly less positive feeling towards life quality in general (Table 4).

**Table 4:** Life quality measures.

		0–3 not happy at all (in %)	4–7 medium happy (in %)	8–10 very happy (in %)	Did not answer (in %)
How happy are you with your life at present?	A	2.8/3.9/1.7	23.6/30.1/17.2	69.9/61.9/78.9	3.7/4.1/3.2
	B	4.9/1.6/1	29.6/23.8/14.7	61.9/69.7/81.6	3.6/4.9/2.7

		0–3 not meaningful at all (in %)	4–7 medium meaningful (in %)	8–10 very meaningful (in %)	Did not answer (in %)
Is your life meaningful?	A	4.2/5.9/2.4	32.8/36.2/29.1	59.1/53.2/65.1	3.9/4.7/3.4
	B	7.5/2.4/1.3	37.2/35.3/24.9	51.2/57.4/71.1	4.1/4.9/2.7

		0–3 did not feel it (in %)	4–7 felt it to some degree (in %)	8–10 felt it very much (in %)	Did not answer (in %)
Last 7 days: felt happy	A	2.7/3.7/1.6	27.2/29.7/24.6	66.2/62.5/69.8	3.9/4.1/4
	B	3.7/2/2	33.7/26.3/18.8	58.5/67.2/76.2	4.1/4.5/3
Last 7 days: felt worried	A	52.6/41.7/63.3	30.6/38.2/22.4	10.4/14.3/6.9	6.4/5.8/7.4
	B	44.3/48.2/67.9	34.3/34/22.4	15.3/10.1/4	6.1/7.7/5.7
Last 7 days: felt down/sad	A	65.3/52.4/78.2	22.4/31.7/12.6	6.4/10.1/2.9	5.9/5.8/6.3
	B	56.8/63.6/78.5	28.4/24.2/12.5	9.7/5.3/3	5.1/6.9/6
Last 7 days: felt irritated	A	45.5/43.4/47.4	39.9/40.8/38.9	8.7/10.2/7.1	5.9/5.6/6.6
	B	42/40.9/54	41/45/34.2	11.7/7.2/6.1	5.3/6.9/5.7
Last 7 days: felt lonely	A	77.7/74.3/81	12.1/14.8/9.1	3.8/4.9/2.7	6.4/6/7.2
	B	73.1/77.7/84.2	15.1/12.8/7	6.2/1.4/2.8	5.6/8.1/6
Last 7 days: felt inspired	A	13.3/16.3/10.3	48.6/50.4/46.8	31.5/27.1/35.5	6.6/6.2/7.4
	B	12.6/11.7/15.8	52.6/48.6/43.3	28.7/31.6/34.9	6.1/8.1/6
Last 7 days: felt calm/relaxed	A	14.6/18.4/10.5	41.1/45.6/36.1	38.6/30.2/47.3	5.7/5.8/6.1
	B	16.7/15.8/10.4	43.2/38.5/40.3	35.2/39.2/42.9	4.9/6.5/6.4
Last 7 days: felt anxious	A	66.9/59.7/74.1	19.7/26.6/14.4	6.3/7.3/3.3	7.1/6.4/8.2
	B	62.1/64.4/75.9	21.7/23.5/13.8	10.1/4/2.6	6.1/8.1/7.7

		0–3 totally disagree (in %)	4–7 medium agreement (in %)	8–10 totally agree (in %)	Did not answer (in %)
My social relations are supportive and giving	A	2.2/2/2.5	25.3/23.8/26.6	67.5/68.6/66	5/5.6/4.9
	B	2.8/1.2/2.3	23.3/31.2/23.1	69.3/62.3/69.2	4.6/5.3/5.4
I contribute to other people's happiness and life quality	A	1.6/2/1.2	27.1/23.6/30.6	66.6/69.3/63.5	4.7/5.1/4.7
	B	1.4/2/1.7	27.8/27.1/26.5	65.5/66/68.1	5.3/4.9/3.7

*Note*: Total number of participants: 957; 485 females and 472 males. Cohorts: 18 YOA = 412, 16 YOA = 247, and 14 YOA = 298. The results are presented in line A; the total number of participants/females/males, line B; 18 YOA/16 YOA/14 YOA, with graded responses from 0 to 10. All numbers are presented as % of the cohort.

Abbreviations: TMJ, temporomandibular joint; YOA, year of age.

In correlation statistics, self‐assessment of general health is significantly related to mental status expressions, showing a positive correlation to positive mental expressions (happiness, meaningfulness, joy, calmness, etc.), and a negative correlation to more negatively loaded mental expressions. General health is significantly correlated to gender, with increased health assessment in males. General health is significantly negatively correlated to pain (facial pain and headache), pain intensity and pain duration, and pain upon oral function, as well as the use of nonprescriptive drugs.

## 4 DISCUSSION

Our study aimed to map the prevalence of TMD among adolescents in Vestfold County, with a focus on myofascial pain, oral function, and quality of life. According to DC/TMD criteria, TMJ noises without symptoms are classified as TMD, but this symptom is rather common and rarely a problem for patients (Bjørnland & Møystad, 2010; Scrivani et al., 2008). These findings were confirmed in our study, where 47.1% of all the participants reported clicking from the TMJ, and in most cases, clicking was not related to neither facial pain nor functional problems. This may indicate that it can be a physiological condition rather than a disease. Asymptomatic clicking may therefore be not relevant in the classification of TMD. At the same time, we found that clicking was significantly more common in the presence of pain. Among the 2% of the adolescents who reported pain upon mouth opening often/quite often, we found that pain upon chewing and TMJ clicking was more frequent.

Concerning oral function, our study showed that 54% of the participants had MIO in a range of 40–50 mm, 36% had MIO above 50 mm, and of those 8% had a range of 60–65 mm. Our findings are supported by other studies (Landtwing, 1978; Müller et al., 2013).

Bjørnland and Møystad (2010) found pain upon palpation of other muscles than the temporal muscle, temporalis, did not necessarily give reduced MIO. Our findings showed that a reduced MIO was significantly associated with higher facial/jaw pain, increased pain during mouth opening and chewing, and pain upon palpation of the masseter muscle. Pain upon palpation of the masseter muscle was significantly associated with limitation of lateral jaw movement to the same side, as well as to the contralateral side as palpated. That mandibular motion usually is limited with TMD, and attempts at active motion, such as chewing, talking, or yawning, increase the pain, are supported by other studies (Bjørnland & Møystad, 2010; Scrivani et al., 2008).

Females experienced facial‐ and TMJ pain more frequently than males, which supports that gender differences are also evident among adolescents (Graue et al., 2016; List & Jensen, 2017; List et al., 1999; Østensjø et al., 2017; Wahlund, 2003). Almost half of the participants had experienced facial pain in the last 3 months, and the observation that headache was the most frequent pain experience, corresponds with the results of other studies (List & Jensen, 2017; List et al., 1999). There was no correlation between headache and mouth function, but the correlation between headache and pain upon mouth opening and chewing was strong. A significantly higher prevalence was found for females compared to males for all pain sites, and facial pain was significantly higher in the oldest age group. The study by Graue et al. (2016) showed a peak of TMD pain at 16 YOA and Østensjø et al. (2017) showed a median age at 17 YOA, whereas our study indicated that pain is more frequent in the 18 YOA group.

In our study, 57% of participants reported the use of nonprescription painkillers, highest among females, and in the oldest age cohort, with nonfeverish headache as the main indication. High consumption of nonprescription painkillers may indicate a lower life quality among adolescents. The use of over‐the‐counter (OTC) analgesics in adolescents has increased, and among 15–16‐year‐olds, 50% of boys and 71% of girls have used it in the last month (Lagerløv et al., 2009). Adolescents, who frequently use OTC analgesics, suffer more pain and have more complex life problems. Their ability to handle stress appears to be discordant with their stressful experiences, and their pain experience seems to be heightened (Skarstein et al., 2014). Findings suggest that OTC analgesics use is common among adolescents to treat pain and other nonmedically indicated conditions, such as stress and anxiety (Kiza et al., 2021). Low use of OTC analgesics may suggest a higher satisfaction with life and less mental stress and better management of pain.

Related to general health, our results were significantly negatively correlated to pain (facial pain and headache), pain intensity and pain duration, and pain upon oral function, as well as the use of nonprescriptive drugs. This differs from the results of Wahlund (2003) and Al‐Khotanis et al. (2016), where TMD was strongly connected with stress, somatic complaints, and emotional problems in adolescents. Wahlund (2003) also found that adolescents with TMD‐related pain reported higher consumption of analgesics in contrast to our study.

Most adolescents in our study were happy with their lives at present and found life meaningful but gender and age differences were observed. The results of our study indicated that about twice as many females felt worried, anxious, and lonely, and just over three times felt sad, as compared to men. Surprisingly, although females in the oldest age group expressed significantly less positive feelings towards life quality in general, contribution to other people's happiness and life quality was higher than for males.

No participants in this study reported pain from TMJ, spontaneously or upon palpation. Surprisingly, there is neither any reduced movement of the jaw in the group with the highest pain related to mouth opening, chewing, and clicking from TMJ compared to the remaining participants nor any differences comparing the use of nonprescription painkillers. Adolescents in this group reported good general health, good life quality, and meaningful life. These findings differ from other studies which show that TMD and pain can be associated with reduced general health, depression, and other psychological disabilities (Al‐Khotani et al., 2016; Bjørnland & Møystad, 2010; Gauer & Semidey, 2015; Haraldstad et al., 2017; List & Jensen, 2017; Wahlund, 2003). Haraldstad et al. (2003) also found that children with pain are generally less happy and have a less positive perception of themselves and are less satisfied with their relationship with their parents.

An important part of this study was to increase the level of expertise on facial‐ and TMJ pain in the dental health service in Vestfold County, and clarify which clinical examinations related to bite physiology should be included in the dental recall examination at the clinic.

The practitioners should have sufficient knowledge of which facial‐ and TMJ pain they can treat and when a patient should be referred to a specialist for follow‐up, so the patient feels cared for and does not become a pawn in the system due to a lack of knowledge of the part of the practitioner. With increased knowledge and focus on facial‐ and TMJ pain, practitioners will be better equipped to identify and follow up with patients with these complaints. This study has only dealt with the identification of facial‐ and TMJ pain, with no follow‐up of the condition.

General practitioner (GP) will also risk coming across patients with facial‐ and TMJ pain, good cooperation between the GP and the dental health service is therefore important for good care of these patients.

There are some limitations in our study. There are many examiners, which increases the vulnerability to reproduce an identical examination of the patient. Conducting clinical examinations according to the DC/TMD protocol is time‐consuming, and this was a solution to get a larger sample within the existing logistical and financial limits. To ensure the most identical examination possible, all examiners were thoroughly calibrated as described under “Design and settings.”

For participants below 16 years old, written consent had to be signed by their parents or guardian, and then either be sent by post or brought to the clinic to join the study. There would presumably be more participants if there was an option to send the signed consent digitally.

The clinical examination was based on DC/TMD when performing muscle palpation, but we did not investigate whether the patients knew if there was “familiar pain.”

## 5 CONCLUSION

In conclusion, we found that almost half of the participants had experienced facial pain in the last 3 months, and the headache was the most frequent pain experience followed by pain upon chewing and mouth opening. A significantly higher prevalence was found for females compared to males for all pain sites, and facial pain was significantly higher in the oldest age group. A reduced MIO was significantly associated with higher reported facial/jaw pain, with increased pain upon chewing and mouth opening. General health was found to be negatively correlated to facial pain, headache, pain intensity, and duration, and pain upon oral function, as well as the use of nonprescriptive drugs. Females in the older age group, experienced less quality of life in general, as they felt more worried, anxious, lonely, and sad, compared to males.

Dentists and dental hygienists have an important role in identifying and diagnosing facial‐ and TMJ pain. Our study may contribute to early identification and treatment/follow‐up and thereby reduce the risk of developing chronic pain among adolescents and reduce its significance in lowering life quality. From a wider perspective, this may result in a socioeconomic benefit by preventing future health problems in adults.

A strength of our study is the large number of participants measuring MIO and lateral movement of the mandible in adolescents. Therefore, our study may be used as a standard in further research on mandibular function in adolescents.

## AUTHOR CONTRIBUTIONS

All the authors have made substansial contributions to the design, analyses and interpretation of data. They have also been involved in drafting the manuscript and revising it. Furthermore all have given final approval for publishing the manuscript and agreed to be accountable for all aspects of the work.

## CONFLICT OF INTEREST STATEMENT

The authors declare no conflict of interest.

## Supporting information

Supporting information.

## Data Availability

Data are available on request due to privacy/ethical restrictions. The data that support the findings of this study are available on request from the corresponding author. The data are not publicly available due to privacy or ethical restrictions.
