# Supplementary material for: Temporomandibular pain and quality of life assessment in adolescents in a Norwegian cohort
Source: Clin Exp Dent Res. 2023 May 26;9(3):481–90. doi: 10.1002/cre2.733 (PMC10280601; doi:10.1002/cre2.733)
Supplement: Supplementary file 1 — Supporting information. [file CRE2-9-481-s001.docx]

Dr. Andreas Stavropoulos

Editor in Chief, Clinical and Experimental Dental Research

**Resubmission of manuscript, ID CRE2.20230002 entitled "Temporomandibular pain and Quality of life assessment in adolescents in a Norwegian cohort”.**

I hereby forward our manuscript for review and possible publication in Journal of Oral Rehabilitation, on behalf of myself and my co-authors, Anne Fykerud Dahl, Anne Kristine Bergem and Heming Olsen-Bergem.

We want to thank you and the referees for valuable and constructive comments to our manuscript. We have made changes in the manuscript according to the referees’ suggestions. Our reply to the referees’ comments, including point to point responses to each of the comments is written in *“Italic types”* after the referees’ comments below. A version of the revised manuscript with the added text highlighted in yellow is downloaded in Manuscript Central in addition to a clean copy without highlighting.

Reviewer(s)' Comments to Author:

Reviewer: 1

Comments to the Author

The Authors have studied TMJ pain and Qol in a population of Norwegian adolescents. As such it adds to the epidemiolocal literature on this subject.

The ms. would benefit from revision for spelling, grammar and style.

*Response: We have carefully revised the manuscript with regard to spelling, grammar and style and the changes are highlighted in the text.*

Abbreviations ie MIO should be used the first time the phrase is mentioned.

*Response: We agree and have changed it in the abstract and text page 7.*

The Authors refer to similar studies conducted in other Norwegian Counties. Can they compare and contrast these studies and is Vestfold Co urban or rural and what are its other socioeconomic characteristics?

*Response: We agree and have and socioeconomic characteristics about Vestfold on page 5.*

They state that MIO was higher than anticipated. Why is that surprising-please explain why MIO is expected to be lower in adolescents.

*Response: We agree and have changed the introduction to more relevant studies about maximal mouth opening capacity on page 3, and on page 13.*

General health did not correlate with Orofacial pain in this study. Why not?

*Response: We may only speculate upon this, but general health was mainly good in this cohort. We may have seen correlation if we had more patients with more severe health problems.*

Given the healthcare system in Norway how will these results be used to improve population health? Are there any lessons for other Healthcare systems?

*Response: We agree and have added more about this on page 15 and 16*

A fuller discussion of the strengths and weaknesses of the study is necessary.

*Response: We agree and have added limitation of the study on page 16.*

Reviewer: 2

Comments to the Author

I have now reviewed the manuscript CRE2.20230002 ”Temporomandibular pain and quality of life assessment in adolescents in a Norwegian cohort”.

The aim was to examine the prevalence of TMD pain in adolescents and to contribute to more focus on this patient group. The diagnostic system DC/TMD was used for the clinical examination and the adolescents answered a survey including questions about pain, pain-related disability, emotional functioning and quality of life.

The manuscript presents results from a study among adolescents in three age cohorts. Adolescents were examined according to DC/TMD, but no diagnoses are presented. That would have been more interesting than ”signs and symptoms”. Too much focus has been on mandibular range of movement and that is my main objection, since the aim was to study the prevalence of pain in the face and jaws.

What authors chose to present are ”millimeter-data” on mouth opening capacity and lateral movements (Table 1).

Instead of presenting the pain diagnoses (myalgia, arthralgia, headache attributed to TMD) that one get from DC/TMD, authors present ”pain upon palpation” (Table 3).

Tables 2 and 4 present self-reported data concerning health, oral health, pain, and general mental health. Unfortunately the tables are very difficult to read and thereby to understand. I recommend authors to redo the tables so that they become more relevant and readable.

*Response: We agree with the referee and have changed the tables 2,3 and 4 accordingly. We have also added in the Method part that specific diagnostic accuracy are difficult in this survey of patients. We have also added in the Result part page 11, a reference to Nilsson et al about patients with TMD*

Here are some further comments on the manuscript:

Introduction:

Please try to be more clear in the introduction so the readers can understand if you write about adults or adolescents. There are several other studies on TMD prevalence in adolescents that you could refer to.

*Response: We agree with the referee and have changed the text accordingly on page 3.*

Page 3:

1st paragraph; ”Longstandning TMD without a correct diagnosis…” I find the references you selected to be inadequate. Ref 1 is a cross-sectional study, not longitudinal, so it is difficult to say anything about chronicity. I don´t find that in Ref 3 either.

*Response: We agree with the referee and have deleted the sentence from the introduction*

4th paragraph; ”Studies from the US show…” concerning adults. Ref 1 is from Norway, about adolescents.

*Response:We have deleted the paragraph form the introduction to have less focus on adults.*

5th paragraph; List et al (ref 9) was about children and adolescents – not young adults. Please try to be careful with the age groups – adolescents are up to 19 (according to WHO). Young adults are older than that.

*Response: We agree with the referee that we should have been more precise in the nomenclature and have changed the text to adolescents on page 3*

Page 4:

2nd paragraph; I think you should add that in the first sentence you write about adults, as the references 3-6 refer to adults.

*Response: We agree with the referee and have added in the text page 4, 2^nd^ paragraph: Several studies of the adult population….*

Materials and Methods

Page 5:

All examiners were calibrated – can you describe the calibration process more, how extensive was the calibration? Can you present reliability (Kappa) values?

*Response: We agree with the referee and have added more about the calibration process on page 6.*

Page 6:

Data collection: I think you should describe the quality of life instrument more in detail. M1-M12 etc needs explanation.

*Response: We agree with the referee and have explained this on page 7*

Authors write that the clinical examination was based on DC/TMD, and that the VAS was used to register pain on palpation. In the DC/TMD when performing muscle palpation, the patients are asked about ”familiar pain”. ”Familiar pain” as concept is basic for the DC/TMD diagnostics. Did you ask for familiar pain?

*Response: We did not include this question in this study, but have mentioned it under limitations of the study on page 16*

I found too much focus on jaw movements when the aim was concerning pain in the face and jaws. This I find to be a fundamental problem with this manuscript.

*Response: We agree with the referee and we have included in the aim of the study, page 4, that oral function also was an important aim: “ The aims of this study was to examine the prevalence of pain from the face and TMJ and oral function….”. We have also added “..and oral function” to Objective on page 2.*

Results

Authors use the DC/TMD to diagnose the adolescents with muscle or joint pain. What is presented in Table 3 has nothing to do with DC/TMD. Furthermore the table is very difficult to read, too many numbers, not clear enough.

*Response: We have corrected Table 3*

Discussion:

Too much focus in mouth opening and lateral movements, not so interesting and not new. I don´t find your results concerning mouth opening surprising. Müller et al. 2013 examined 20,719 children and adolescents with similar findings.

I think you should hold on to your title and concentrate on TMD pain and quality of life assessment.

*Response: We agree and have changed the text about our MIO results on page 12 and 13.*

Sincerely yours,

Tore Bjørnland
